# Production of synthetic wheat lines to exploit the genetic diversity of emmer wheat and D genome containing *Aegilops* species in wheat breeding

**DOI:** 10.1038/s41598-020-76475-7

**Published:** 2020-11-12

**Authors:** Ghader Mirzaghaderi, Zinat Abdolmalaki, Rahman Ebrahimzadegan, Farshid Bahmani, Fatemeh Orooji, Mohammad Majdi, Ali-Akbar Mozafari

**Affiliations:** 1 Department of Agronomy and Plant Breeding, Faculty of Agriculture, University of Kurdistan, P. O. Box: 416, Sanandaj, Iran; 2 Department of Horticultural Sciences, Faculty of Agriculture, University of Kurdistan, P. O. Box: 416, Sanandaj, Iran

**Keywords:** Plant sciences, Plant breeding

## Abstract

Due to the accumulation of various useful traits over evolutionary time, emmer wheat (*Triticum turgidum* subsp. *dicoccum* and *dicoccoides*, 2*n* = 4*x* = 28; AABB), durum wheat (*T. turgidum* subsp. *durum*, 2*n* = 4*x* = 28; AABB), *T. timopheevii* (2*n* = 4*x* = 28; AAGG) and D genome containing *Aegilops* species offer excellent sources of novel variation for the improvement of bread wheat (*T. aestivum* L., AABBDD). Here, we made 192 different cross combinations between diverse genotypes of wheat and *Aegilops* species including emmer wheat × *Ae. tauschii* (2*n* = DD or DDDD), durum wheat × *Ae. tauschii*, *T. timopheevii* × *Ae. tauschii*, *Ae. crassa* × durum wheat, *Ae. cylindrica* × durum wheat and *Ae. ventricosa* × durum wheat in the field over three successive years. We successfully recovered 56 different synthetic hexaploid and octaploid F_2_ lines with AABBDD, AABBDDDD, AAGGDD, D^1^D^1^X^cr^X^cr^AABB, D^c^D^c^C^c^C^c^AABB and D^v^D^v^N^v^N^v^AABB genomes via in vitro rescue of F_1_ embryos and spontaneous production of F_2_ seeds on the F_l_ plants. Cytogenetic analysis of F_2_ lines showed that the produced synthetic wheat lines were generally promising stable amphiploids. Contribution of D genome bearing *Aegilops* and the less-investigated emmer wheat genotypes as parents in the crosses resulted in synthetic amphiploids which are a valuable resource for bread wheat breeding.

## Introduction

*Triticum urartu* Tumanian ex Gandilyan (2*n* = 2*x* = 14, genome AA) and a species from section *Sitopsis*, most likely *Aegilops speltoides* Tausch (2*n* = 2*x* = 14, genome SS) are the A and B genome progenitors of emmer wheat (*T. turgidum*)^1–3^. The ancestors of these species naturally hybridised about 0.36–0.5 million years ago to create the most ancient polyploid wheat: wild emmer wheat (*T. turgidum* subsp. *dicoccoides* (Korn. ex Asch. & Graebn.) Schweinf)^4,5^. Many useful traits are known to be present in emmer wheat, making it a particularly important source of exotic disease resistance genes and for end-use quality, drought tolerance and yield improvement of bread wheat. Cultivated emmer wheat (*T*. *turgidum* L. subsp. *dicoccon* Schrank; syn. *T. turgidum* L. subsp. *dicoccum* Schübl., 2*n* = 4*x* = 28, AABB) was domesticated about 10,000 years ago from its wild emmer wheat progenitor^6^. At that time, natural hybridisation between cultivated emmer and goat grass *Ae. tauschii* Coss. (2*n* = 2*x* = 14, DD) led to the emergence of common wheat (*T. aestivum* L., 2*n* = 6*x* = 42, AABBDD)^7^.

Common wheat suffers from low genetic variation due to the recent foundation of bread wheat from one or a limited number of hybridization events, and from subsequent domestication and selection activities^8–10^. New, distinct varieties of wheat need to be continuously released in response to changing environmental conditions and pathogen evolution to overcome resistances and climate change. Wheat breeding has historically relied on intra and interspecific hybridization to provide new variation and to improve the bread wheat germplasm pool^8,11^. Due to the recent origination of bread wheat, the D subgenome of bread wheat is still substantially similar to the D genome of *Ae. tauschii*, such that introgression of D genome chromosome segments from *Ae. tauschii* into the wheat background does not result in significant deleterious genetic drag in hybrids: as a result, *Ae. tauschii* has been efficiently utilized for the improvement of common wheat for decades^12^.

Useful traits such as tolerance to cold^13^ and salt^14^, leaf and stem rust resistance^15^ and resistance to cereal cyst and root-knot nematodes^16,17^ also exist within of the allopolyploid *Aegilops* species containing a copy of the D genome: *Ae. crassa* 4*x* (2n = D^1^D^1^X^cr^X^cr^), *Ae. crassa* 6*x* (2*n* = D^1^D^1^X^cr^X^cr^D^cr^D^cr^), *Ae. cylindrica* (2*n* = D^c^D^c^C^c^C^c^), *Ae. vavilovii* (2*n* = D^1^D^1^X^cr^X^cr^S^v^S^v^), *Ae. ventricosa* (2*n* = D^v^D^v^N^v^N^v^) and *Ae. juvenalis* (2*n* = D^j^D^j^X^j^X^j^U^j^U^j^). These allopolyploids have largely remained unexploited probably because of crossing barriers in hybridization with bread wheat, deleterious genetic drag or lack of precise molecular techniques to discriminate between the exotic and bread wheat D-genome chromosomal segments (reviewed in Mirzaghaderi and Mason^18^). Among these, *Ae. cylindrica* may be the most recalcitrant species to give amphiploids when hybridized with wheat probably due to high rates of or complete hybrid sterility^19–21^. With the recent achievements in whole genome sequencing such as longer read sequencing technologies and better assembly algorithms, a massive wheat genomic resources has been available, allowing a more efficient introgression of useful phenotypic traits from the D-genome containing species into bread wheat. This can be achieved via crossing of *Aegilops* with wheat to produce amphiploids and subsequent crossing of the resulting materials to bread wheat^22^. However, crossing between *T. turgidum* and *Aegilops* species usually involves barriers that in most cases require embryo rescue to overcome for the subsequent development of synthetic wheat lines^23^.

Here, we aimed to generate novel genetic resources by incorporating genetic diversity of D-genome containing *Aegilops* species including *Ae. tauschii*, *Ae. crassa*, *Ae. cylindrica* and *Ae. ventricosa* and *T. turgidum* and *T. timopheevii* genotypes. For these, we crossed a set of diverse *T. turgidum* (subsp. *dicoccum*, *dicoccoides* and *durum*) and *T. timopheevii* with D genome bearing *Aegilops* species in the field over three successive years. Fifty six different synthetic hexa- and octaploid F_2_ lines were recovered with AABBDD, AABBDDDD, AAGGDD, D^1^D^1^X^cr^X^cr^AABB, D^c^D^c^C^c^C^c^AABB or D^v^D^v^N^v^N^v^AABB genome complements; a subset of these lines were further analyzed by fluorescence in situ hybridization (FISH). Contribution of a tetraploid accession of *Ae. tauschii* (2*n* = 4*x* = 28; DDDD), other D genome containing *Aegilops* species and the less investigated emmer wheat genotypes in the crosses provide novel, useful germplasm that can be used to broaden the bread wheat genetic variation beyond its current status.

## Materials and methods

### Plant material

Eleven different emmer wheat landraces were collected from villages in the Kurdistan province of Iran, and 12 emmer wheat genotypes were received from the Seeds and Plant Improvement Institute of Iran (SPII). An accession of *T. timopheevii* and some of the durum wheat genotypes were received or from The International Center for Agricultural Research in the Dry Areas (ICARDA). The other durum and domesticated emmer wheat genotypes and landraces were received from The International Maize and Wheat Improvement Center (CIMMYT) or Dry Land Agricultural Research Institute (DARI, Maragheh, Iran). *Ae. tauschii*, *Ae. crassa* and *Ae. ventricosa* genotypes were received from the IPK gene bank in Germany, except for ‘G 276’, ‘G 299’, and ‘G 307’ genotypes of *Ae. tauschii* and cultivars of common wheat, which were received from the Seeds and Plant Improvement Institute of Iran. ‘Bookan’ and ‘Sanandaj’ accessions of *Ae. crassa*, ‘1’ and ‘236’ accessions of *Ae. cylindrica* and ‘Hawraman’ and ‘Seysaleh’ ecotypes of wild emmer wheat were collected from North-West regions of Iran. Details about the plant material including subspecies, accession number and origin has been presented in Supplementary Spreadsheet S1.

### Crossing

Crosses between tetraploid wheat genotypes as the female parents and *Ae. tauschii* genotypes as the male parents and crossing between tetraploid *Aegilops* species (*Ae. crassa*, *Ae. cylindrica* and *Ae. ventricosa*) as female parents and tetraploid wheat genotypes as male parents were made by hand between the months of May and June in 2017, 2018 and 2019 at the research farm of the University of Kurdistan. Approximate temperature and humidity during the crossing period ranged from 18 to 37 °C during the day and 5–17 °C at night, with low humidity and precipitation. Only the two outermost florets of spikelets were pollinated. No hormone treatment was applied. The spikes of wild emmer (*T. dicoccoides*) and tetraploid *Aegilops* species were bagged after crossing in order to prevent spikelet dispersal and to enable seed collection.

In late summer, the embryos from the dried mature shriveled F_1_ seeds belonging to each cross combination between *Ae. tauschii* and tetraploid wheat genotypes were rescued. For this, the shriveled seeds were firstly sterilized in 5% sodium hypochlorite solution for 15 min with shaking, rinsed in sterilized distilled water for 2 × 10 min and kept in sterilized distilled water overnight at 4 °C. The seed coat was carefully removed, the embryo was placed on ½ MS media (pH 5.8, including vitamins)^24^ in a sterile jar and maintained under photoperiod of 16 h of light and 8 h of darkness at 22–24 °C. Grown seedlings of about 10 cm long were washed to remove the media and transferred to soil in small pods. The established seedlings were finally transplanted to the field. Chemical treatment for chromosome doubling was not applied, and the production of F_2_ seeds from F_1_ plants in the upcoming spring was relied on the ability of the hybrids to form unreduced male and female gametes.

Hybrid seeds between tetraploid *Aegilops* species and wheat genotypes had endosperm and did not require embryo rescue. In the spring of the following year, non-hybrid plants were weeded out at the flowering stage and only the true hybrid plants—which were morphologically distinguishable—were retained in the field. The F_1_ spikes were bagged to enforce self-pollination. As a rule to indicate the cross direction or genome designation of each hybrid or amphiploid in the present study, the female parent or maternal genome is listed first followed by the male parent or paternal genome.

### Pollen viability analysis

Pollen viability was calculated as the percentage of pollen stained with Alexander's solution^25^. Immature anthers were randomly selected from four spikelets of different tillers in each hybrid combination and 10 anthers were analyzed to measure the percentage of viable pollen grains in each F_1_ cross combination. Strongly stained swollen pollen grains were assumed to be viable.

### Fluorescence in situ hybridization (FISH)

Seed germination, root tip pretreatment and digestion, slide preparation and subsequent FISH experiments were done according to Abdolmalaki et al.^26^. p*Ta*535-1 oligonucleotide probes (5′-AAA AAC TTG ACG CAC GTC ACG TAC AAA TTG GAC AAA CTC TTT CGG AGT ATC AGG GTT TC-3′)^27,28^, and (GAA)10 microsatellite sequences were 5′-end labelled with 6-carboxytetramethylrhodamine (TAMRA) and 6-carboxyfluorescein (FAM), respectively. Probes were synthesized by Bioneer Co. Ltd. (Daejeon, Korea), diluted using 1 × TE solution (pH 7.0) and used at the concentration of 30–50 ng per 20 µl hybridization buffer for each slide in the FISH experiment. After hybridization and washing, slides were dehydrated in ethanol series, dried at RT and counterstained with a drop of Vectashield mounting medium (Vector Laboratories) containing 1 µg/ml DAPI (4′,6-diamidino-2-phenylindole). Slides were inspected with an epifluorescence Olympus BX51 microscope and images were captured using a DP72 digital camera. *T. timopheevii* chromosomes were identified according to Badaeva et al.^29^. *Ae. crassa* chromosomes were identified based on Abdolmalaki et al.^26^. The chromosomes of the C^c^ and D^c^ subgenome of *Ae. cylindrica* were identified according to Mirzaghaderi et al.^30^ and its C^c^ subgenome chromosomes were numbered based on Danilova et al.^31^.

The number of seeds used for cytogenetic works was case-dependent: chromosome analysis of the F_1_ seeds/embryos was done using the root tips of single seeds/seedlings separately and the corresponding seed/seedling was replanted to recovery and grow. For the analysis of the parental lines, three different seeds were commonly used from each line. FISH analysis and chromosome counting of *Ae. cylindrica*-*T. durum*, *Ae. ventricosa*-*T. durum* and *Ae. crassa-T. durum* amphiploids were applied using the single seed roots.

### C-banding

The C-banding technique described by Gill et al.^32^ was used with mirror modifications in slide preparation. Slides were prepared as for FISH and stored in 96% ethanol at − 20 °C for at least 24 h. Slides were then dried at room temperature and used for C-banding.

### Phenotypic evaluation

Amphiploid lines were grown in autumn each in a row at the research farm of the University of Kurdistan main campus. Rows were 5 m long and 0.5 m apart with a sowing rate of 20 seeds per row. The field was watered by both rainfall and irrigation but no fertilizer was applied. Nine representative plants from each line were used for phenotypic evaluation at maturity. The plant height and spike length (both excluding awns), awn length, total spikelets per spike, nodes number, flag leaf width and length, flowering time (from the first day of the spring) and peduncle length were measured from the main tillers.

For the analysis of the iron (Fe) and zinc (Zn) contents, one gram grain samples were digested in a mixture of concentrated HNO_3_ (two parts) and HCl (one part) according to Zarcinas et al.^33^ until a white residue was obtained. The required volume was made up after completion of the digestion process, and digests were analyzed using an atomic absorption spectrophotometer (GBC 902 AA, Australia). Three biological replications from each amphiploid line were analyzed. Fe and Zn concentrations were presented in microgram per gram dry weight of seed (µg/g DW).

### Statistical analysis

The crossability of each parental genotype was calculated as the percentage of the F_1_ embryos or seeds obtained over the total florets pollinated for that cross. Bar graphs of the crossability rates were prepared in the base package of R version 3.6.1 (The R Project for Statistical Computing, Vienna, Austria). Pollen viability data between the cross combinations were analyzed based on completely randomized design in R where the assessed anthers in each cross combination were considered as replications. Because the pollen viability rates in cross combinations were correlated with variance, logarithm of the data were used for the analysis of variance (ANOVA). Pearson’s correlation coefficient was used to demonstrate whether F_1_ seed set rate is correlated with viable gamete rate. Principal component analysis of morphological data were performed in R based on data of morphological traits.

## Results

**Figure 1:**
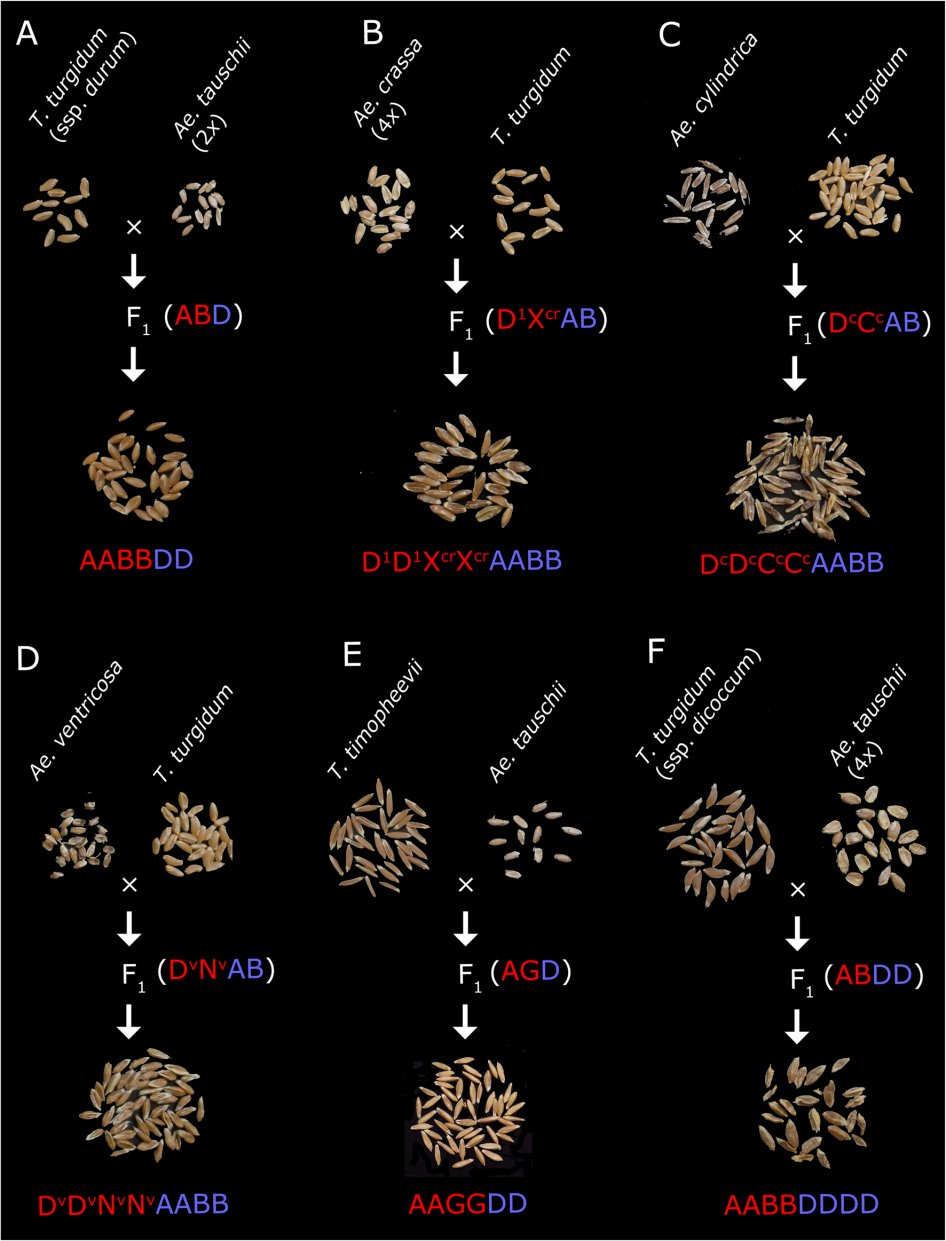
Crossing schemes followed in the present study. (**A**) Crossing using *Ae. tauschii* as male parent for production of synthetic wheat lines. *Ae. crassa* (**B**), *Ae. cylindrica* (**C**) and *Ae. ventricosa* (**D**) were usend as female parents in crossing with *T. turgidum* to produce F_1_ hybrids and amphiploids. (**E**) Crossing using *T. timopheevii* as female parent and *Ae. tauschii* as male parent. (**F**) Crossing of *T. turgidum* as female parent with a tetraploid *Ae. tauschii* as male parent. Seed pictures of the parents and the amphiploids are shown for each cross.

192 different cross combinations between diverse genotypes of emmer wheat × *Ae. tauschii* (2*n* = DD or DDDD), durum wheat × *Ae. tauschii*, *T. timopheevii* × *Ae. tauschii*, *Ae. crassa* × durum wheat, *Ae. cylindrica* × durum wheat and *Ae. ventricosa* × durum wheat were made in the field over three successive years. We successfully recovered 56 different synthetic hexaploid and octaploid F_2_ lines with AABBDD, AABBDDDD, AAGGDD, D^1^D^1^X^cr^X^cr^AABB, D^c^D^c^C^c^C^c^AABB and D^v^D^v^N^v^N^v^AABB genomes via in vitro rescue of F_1_ embryos and spontaneous production of F_2_ seeds on the F_l_ plants. The crossing schemes and corresponding seed morphology of parental species and resulting amphiploids is shown in Fig. 1.

### Phenotypic diversity in the parent lines

**Figure 2:**
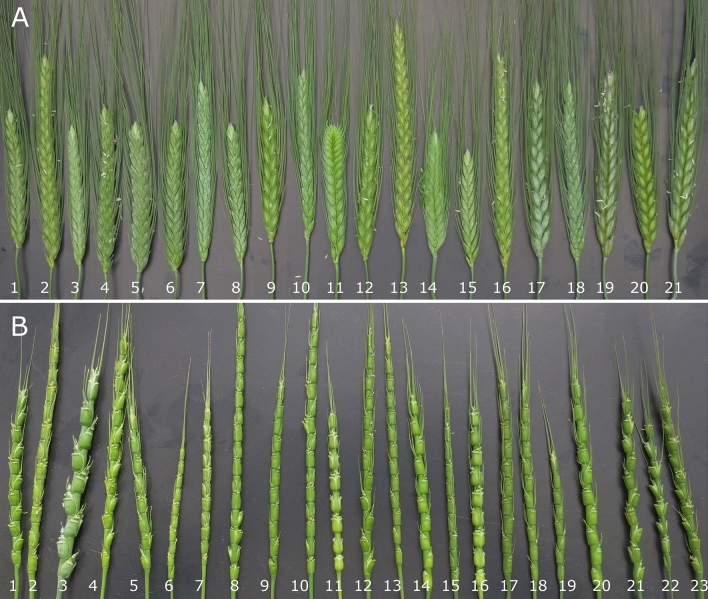
Spike morphology of emmer wheat (**A**) and *Ae. tauschii* (**B**) genotypes used for crossing in the present study. Emmer accessions: (1) Kalakan, (2) 49662, (3) Tirgaran, (4) 49659, (5) TazeabadAliabad, (6) Tarkhanabad, (7) IG 127691, (8) Chatan, (9) Hawraman, (10 IG 88753, (11) IG 88732, (12) Seysaleh, (13) 49663, (14) 49657, (15) Arandan, (16) 49666, 17) IG 88882, (18) IG 127687, (19) 49664, (20) 49661, (21) 49665. *Ae. tauschii* accessions: (1) AE 277, (2) AE 3, (3) AE 1211, (4) AE 1650, (5) AE 955, (6) AE 1067, (7) AE 1055, (8) G 276, (9) AE 964, (10) G 307, (11) AE 1600, (12) AE 142, (13) AE 3675, (14) AE 191, (15) AE 235, (16) AE 1602, (17) AE 13938, (18) AE 956, (19) AE 167, (20) AE 143, (21) AE 1037, (22) AE 596, (23) AE 541.

Emmer wheat and *Ae. tauschii* genotypes showed highly variable spike morphologies (Fig. 2). A high level of diversity overall was found among the parental emmer wheats for most morphological traits measured. Diversity was especially high for flag leaf width (5.6–19.6 mm), spikelets per spike (10.3–29.6; can also be seen in Fig. 2), spike length (6.1–13.6), flowering time (44–93 days from the first of the spring), seed Fe (24.2–66.3 µg/g DW) and Zn (16–62.3 µg/g DW) contents under no fertilizer conditions. Most emmer wheat landraces collected from the Kurdistan province grouped together based on phenotypic traits (Supplementary Fig. S2). Of these, some genotypes had high Fe and Zn contents in the seed: ‘Bainjub’, ‘Tirgaran’, ‘TazeabadAesef’, ‘Arandan’ and ‘Hawraman’ (Fig. 2, Supplementary Figs. S1 and S2, Supplementary Spreadsheet S2).

### Crosses between tetraploid wheat and *Ae. tauschii*

**Figure 3:**
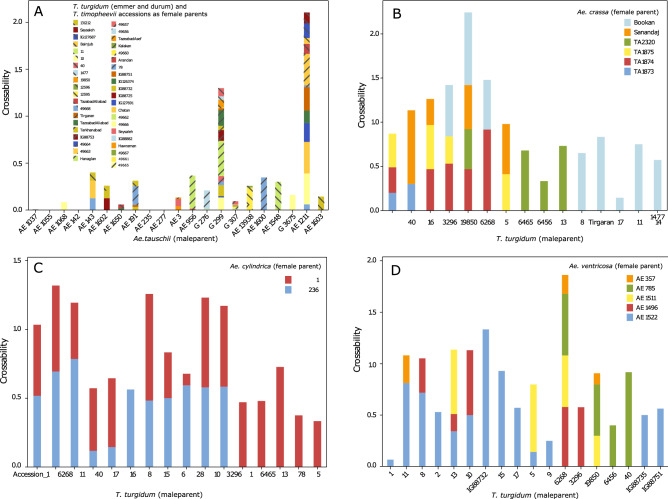
Crossability of *T. turgidum* and *Aegilops* genotypes based on the F_1_ seeds per total pollinated florets. (**A**) Each *Ae. tauschii* genotype was crossed as male parent with *T. timopheevii* (accession 131212) or different *T. turgidum* accessions (as female parents) and the corresponding cumulative crossability rates are indicated in a single stacked column. (**B**–**D**) Crossability rates of *T. turgidum* accessions as male parent with *Ae. crassa*, *Ae. cylindrica* and *Ae. ventricosa* accessions, respectively.

Emmer wheat and *Ae. tauschii* genotypes as well as one genotype of *T. timopheevii* were used in 105 different cross combinations (101 cross combinations between *T. turgidum* and *Ae. tauschii* plus four cross combinations between *T. timopheevii* and *Ae. tauschii*). Crossability of *T. turgidum* and *Aegilops* genotypes based on the F_1_ seeds per total pollinated florets is shown in Fig. 3. Crossing success between tetraploid wheat and *Ae. tauschii* was relatively moderate, but production of viable hybrids mostly required embryo rescue. Mean crossabilities in *T. turgidum* × *Ae. tauschii* was 0.062, ranging from 0 to 0.38. Of the *Ae. tauschii* genotypes, accessions ‘AE 1211’ and ‘G 299’ showed the highest mean crossability of 0.15 and 0.08 both with emmer wheat genotypes, respectively (Fig. 3A). However, a similar set of emmer wheat couldn’t be crossed with each *Ae. tauschii* genotype due to differences in flowering time of both *Ae. tauschii* and emmer wheat accessions, therefore the genotypic effect might also be involved. The range of flowering time was especially high for emmer wheat (sown in autumn) ranging from 44 to 93 days from the first day of the spring (Supplementary Fig. S1, Supplementary Spreadsheet S2).

From the 101 cross combinations between *T. turgidum* (subsp. *dicoccum* and *dicoccoides* and *durum*) and *Ae. tauschii*, 346 F_1_ seeds were obtained belonging to 44 different cross combinations. Of the 346 F_1_ seeds produced from *T. turgidum* wheat × *Ae. tauschii* crosses, only fourteen had endosperm and could autonomously germinate. These endosperm-containing F_1_ seeds belong to *T. dicoccum* 'Bainjub' × *Ae. tauschii* 'AE 1211’ (2 seeds out of 10), *T. durum* ‘40’ × *Ae. tauschii* 'G 299’ (1 out of 11), *T. durum* '78’ × *Ae. tauschii* 'G 299’ (1 out of 1), *T. durum* ‘78’ × *Ae. tauschii* 'AE 1600’ (2 out of 18), *T. durum* ‘78’ × *Ae. tauschii* 'AE 1211’ (1 out of 2), *T. dicoccum* 'IG88753’ × *Ae. tauschii* 'G 299’ (4 out of 23), *T. dicoccoides* '49660’ × *Ae. tauschii* '13938’ (1 out of 5), *T. dicoccum* 'Kalakan' × *Ae. tauschii* '307’ (1 out of 2) and *T. durum* '1477’ × *Ae. tauschii* 'G 299’ (1 out of 6) crosses. The remaining F_1_ seeds were shriveled or lacked endosperm and hence required embryo rescue (Supplementary Fig. S3). From which, 52% of the F_1_ seeds (47 from 90) were successfully rescued, and resulted plants grown to maturity. F_1_ seeds from 25 cross combinations successfully reached to maturity and produced amphiploid F_2_ seeds (Supplementary Spreadsheet S1). *T. timopheevii* showed a relatively high crossability (0.085 on average) with *Ae. tauschii* and produced 167 thin healthy F_1_ seeds from four cross combinations that could autonomously germinate. By the end, all crosses produced a total of 29 different synthetic hexaploid and octaploid F_2_ lines with AABBDD, AABBDDDD or AAGGDD genomes. The number of obtained F_1_ and F_2_ seeds from these hybrids is shown in Supplementary Spreadsheet S1.

### Crosses between tetraploid *Aegilops* and tetraploid wheat

Different genotypes of D genome containing tetraploid *Aegilops* species (*Ae. crossa*, *Ae. cylindrica* and *Ae. ventricosa*) and tetraploid wheat lines were used in 87 different cross combinations. The number of F_1_ seeds produced per total wheat florets pollinated by each *Aegilops* is shown in Supplementary Spreadsheet S1 and the crossability rates for each cross combinations is indicated in Fig. 3. Crossing success between tetraploid *Aegilops* and tetraploid wheat was relatively high and the F_1_ seeds could autonomously germinate. A total of 262 octaploid F_2_ amphiploid seeds with D^1^D^1^X^cr^X^cr^AABB, D^c^D^c^C^c^C^c^AABB and D^v^D^v^N^v^N^v^AABB genomes were recovered from 27 different cross combinations. Mean crossability rates in crosses between *Ae. crassa*, *Ae. cylindrica* and *Ae. ventricosa* with tetraploid wheat lines were 0.52, 0.49 and 0.43, respectively. The overall mean crossability in all the crosses was 0.51. Similar to wheat-*Ae. tauschii* crosses, a similar set of emmer wheat couldn’t be crossed with each *Aegilops* genotype due to differences in flowering time of both *Aegilops* and wheat accessions. 1322 seeds were produced from crosses between tetraploid wheat and tetraploid *Aegilops* (e.g. *Ae. crossa*, *Ae. cylindrica* and *Ae. ventricosa*). All of these F_1_ seeds had endosperm, but from a sample of 50 F_1_ seeds, 28 (0.56) germinated in Petri dishes. 74% of these germinated seeds resulted plants grown to maturity. The number of obtained F_2_ seeds from these hybrids is shown in Supplementary Spreadsheet S1.

### Pollen viability

**Figure 4:**
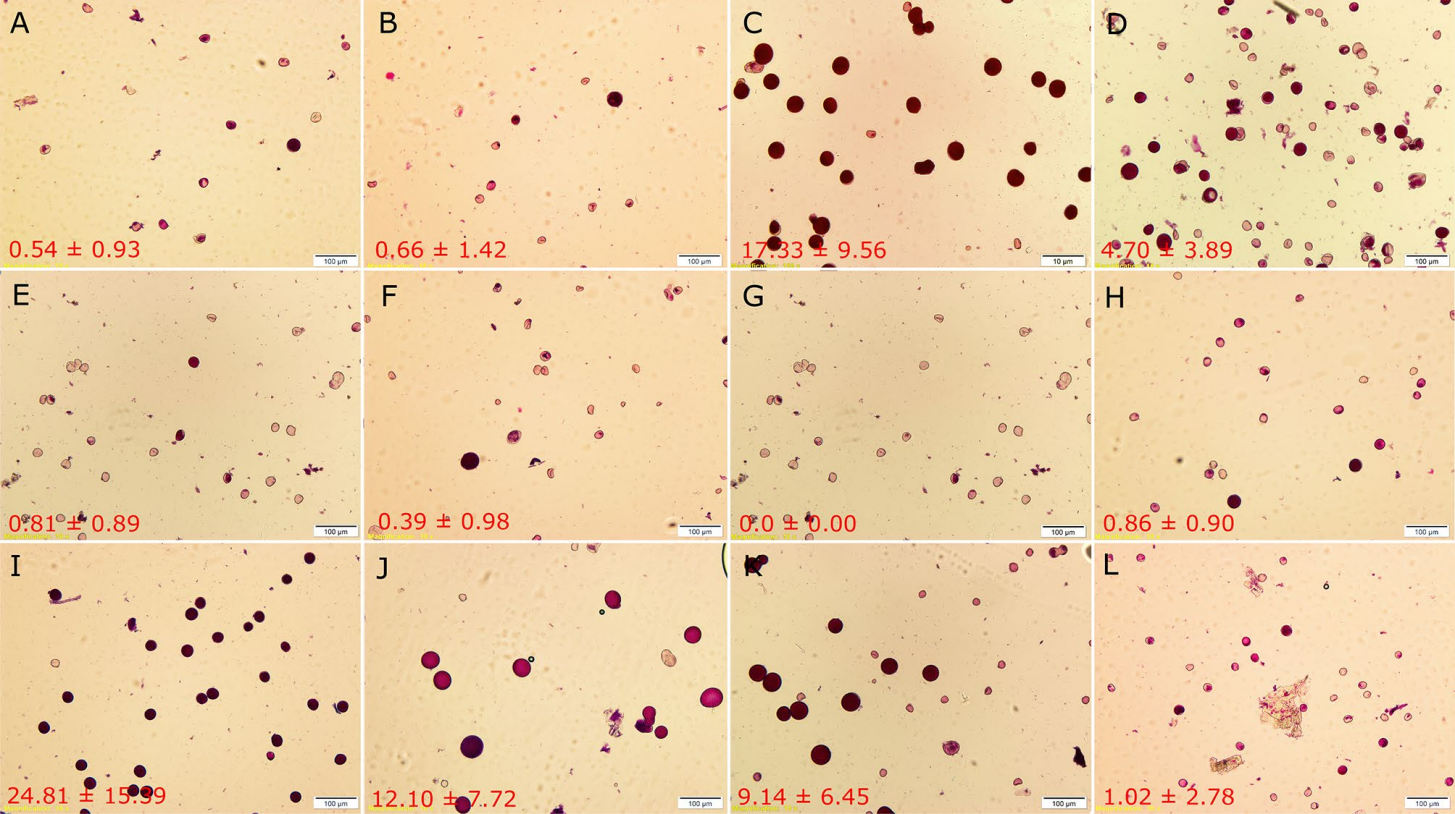
Differential staining of aborted and non-aborted pollen grains using Alexander's stain and percentage of viable pollens (± standard deviation) in hybrid plants: *Ae. crassa* ‘Bookan’ × *T. durum* ‘14’ (**A**), *Ae. crassa* ‘TA1873’ × *T. durum* ‘40’ (**B**), *Ae. crassa* ‘Sanandaj’ × *T. durum* ‘6268’ (**C**), *Ae. crassa* ‘TA1875’ × *T. durum* ‘40’ (**D**), *Ae. crassa* ‘TA1874’ × *T. durum* ‘19850’ (**E**), *Ae. crassa* ‘Bookan’ × *T. durum* ‘6268’ (**F**), *T. durum* ‘17’ × *Ae. cylindrica* ‘236’ (**G**), *T. dicoccum* ‘IG 88753’ × *Ae. tauschii* ‘G 299’ (**H**), *T. dicoccum* ‘IG 12638’ × *Ae. tauschii* ‘AE 1650’ (**I**), *T. dicoccum* ‘IG 127691’ × *Ae. tauschii* ‘G 299’ (**J**), *T. dicoccum* ‘TazabadAliabad’ × *Ae. tauschii* ‘AE 1651’ (**K**), *Ae. ventricosa* ‘AE 1522’ × *T. durum* ‘11’ (**L**). Scale bar = 100 µm.

We assessed pollen viability in six *Ae. crassa* × *T. turgidum*, four different *T. turgidum* × *Ae. tauschii*, one *T. turgidum* × *Ae. cylindrica* and one *Ae. ventricosa* × *T. turgidum* hybrid plants (Fig. 4). The analyzed hybrids varied significantly in pollen viability (F = 18.76***; df_error_ = 108) and plump seed set. Four *T. turgidum* × *Ae. tauschii* hybrids from different cross combinations produced viable pollen grains at 0.86%, 24.81%, 12.10% and 9.14% frequencies on average. Similarly, the mean frequencies of viable pollen grains in the analyzed *Ae. crassa* × *T. turgidum* hybrids were highly variable, varying from 0.39% (for *Ae. crassa* ‘Bookan’ × *T. durum* ‘6268’ hybrid) to 17.33% (for *Ae. crassa* ‘Sanandaj’ × *T. durum* ‘6268’ hybrid). No viable pollen was observed for *T. durum* ‘17’ × *Ae. cylindrica* ‘236’. Mean of unreduced gamete in *Ae. ventricosa* ‘AE 1522’ × *T. durum* ‘11’ hybrid was 1.02%. Unreduced gamete rates were correlated with the rates of plump seed set (*r* = 0.75, *P* = 0.005).

### Chromosomal constitutions of the synthetic wheat lines

**Figure 5:**
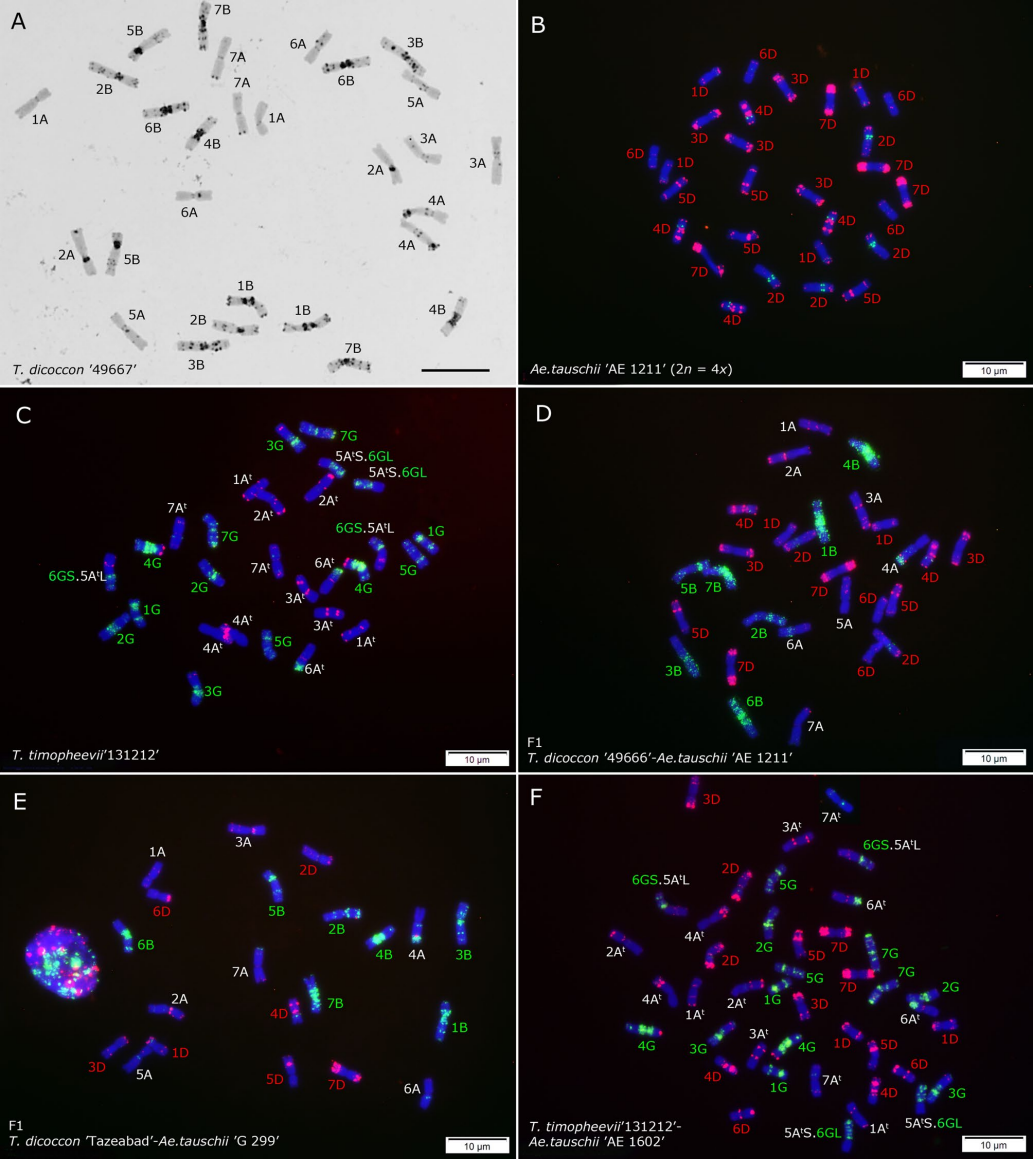
C-banding of mitotic metaphase chromosomes of *T. dicoccum* ‘49667’ (**A**) and FISH on mitotic metaphase chromosomes *Ae. tauschii* ‘AE 1211’ (**B**); *T. timopheevii* ‘131212’ (**C**); an F_1_ seed from a cross between *T. dicoccum* ‘49666’-*Ae. tauschii* ‘AE 1211’ (**D**); an F_1_ seed from a cross between *T. dicoccum* ‘TazeabadAliabad’-*Ae. tauschii* ‘G 299’ (**E**) and an amphiploid from a cross between *T. timopheevii* ‘131212’-*Ae. tauschii* ‘AE 1602’ (**F**). FISH signals are from (GAA)_10_ (green) and p*Ta*535-1 (red) oligonucleotide probes, respectively.

**Figure 6:**
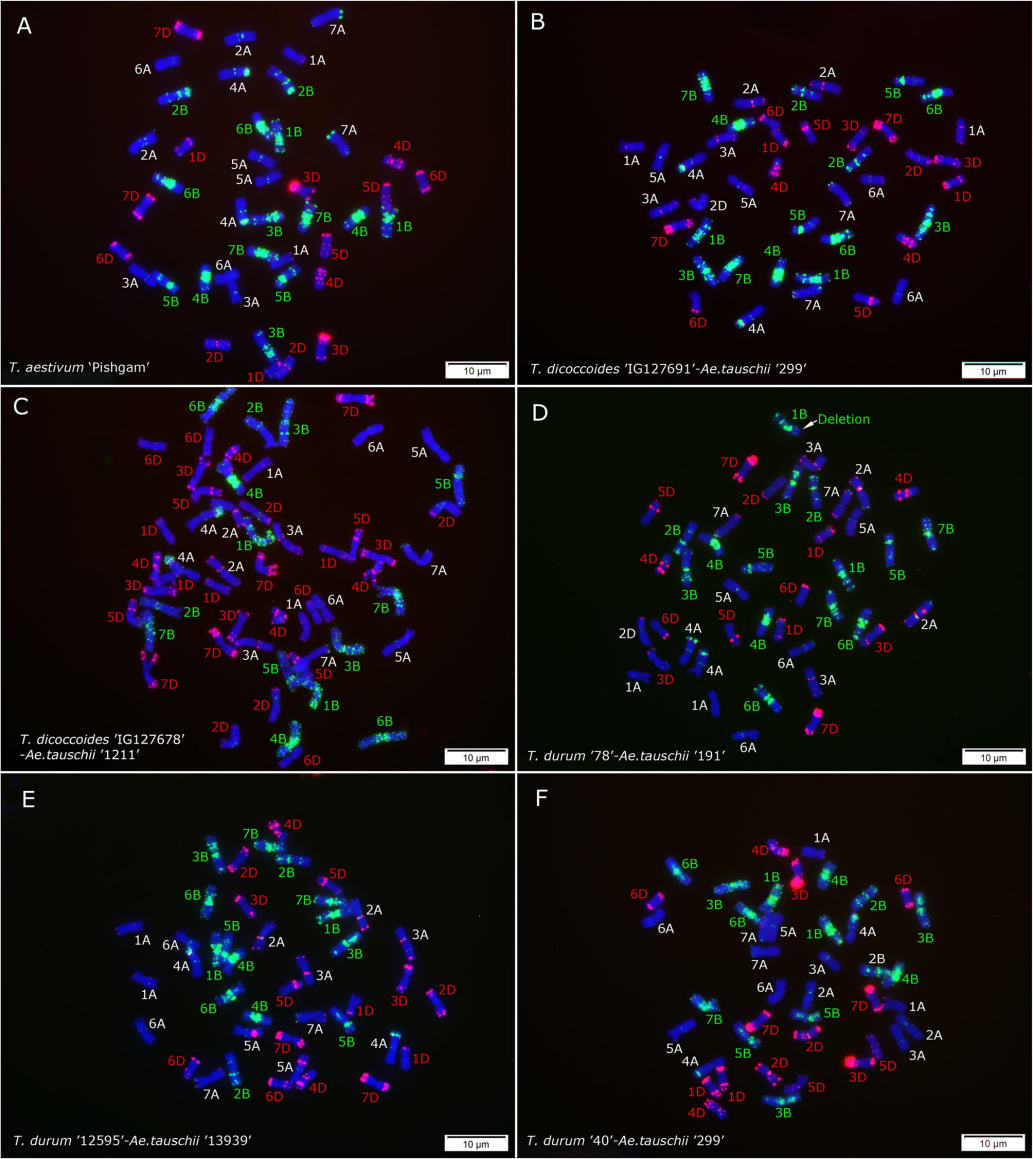
FISH signals from (GAA)_10_ (green) and p*Ta*535-1 (red) probes on mitotic metaphase chromosomes of *T. aestivum* ‘Pishgam’ (**A**) and different synthetic wheat lines (in F_2_) generated from crosses between *T. dicoccoides* ‘IG127691’ × *Ae. tauschii* ‘G 299’ (**B**), *T. dicoccoides* ‘IG127678’ × *Ae. tauschii* ‘AE 1211’ (**C**), *T. durum* ‘78’ × *Ae. tauschii* ‘AE 191’ (**D**), *T. durum* ‘12595’ × *Ae. tauschii* ‘13939’ (**E**) and *T. durum* ‘40’ × *Ae. tauschii* ‘G 299’ (**F**). Synthetic wheat lines show 42 chromosomes except in C where a tetraploid accession of *Ae. tauschii* i.e. ‘AE 1211’ was used in the cross, resulting in an amphiploid with 56 chromosomes. Chromosomes were counterstained by DAPI (blue).

**Figure 7:**
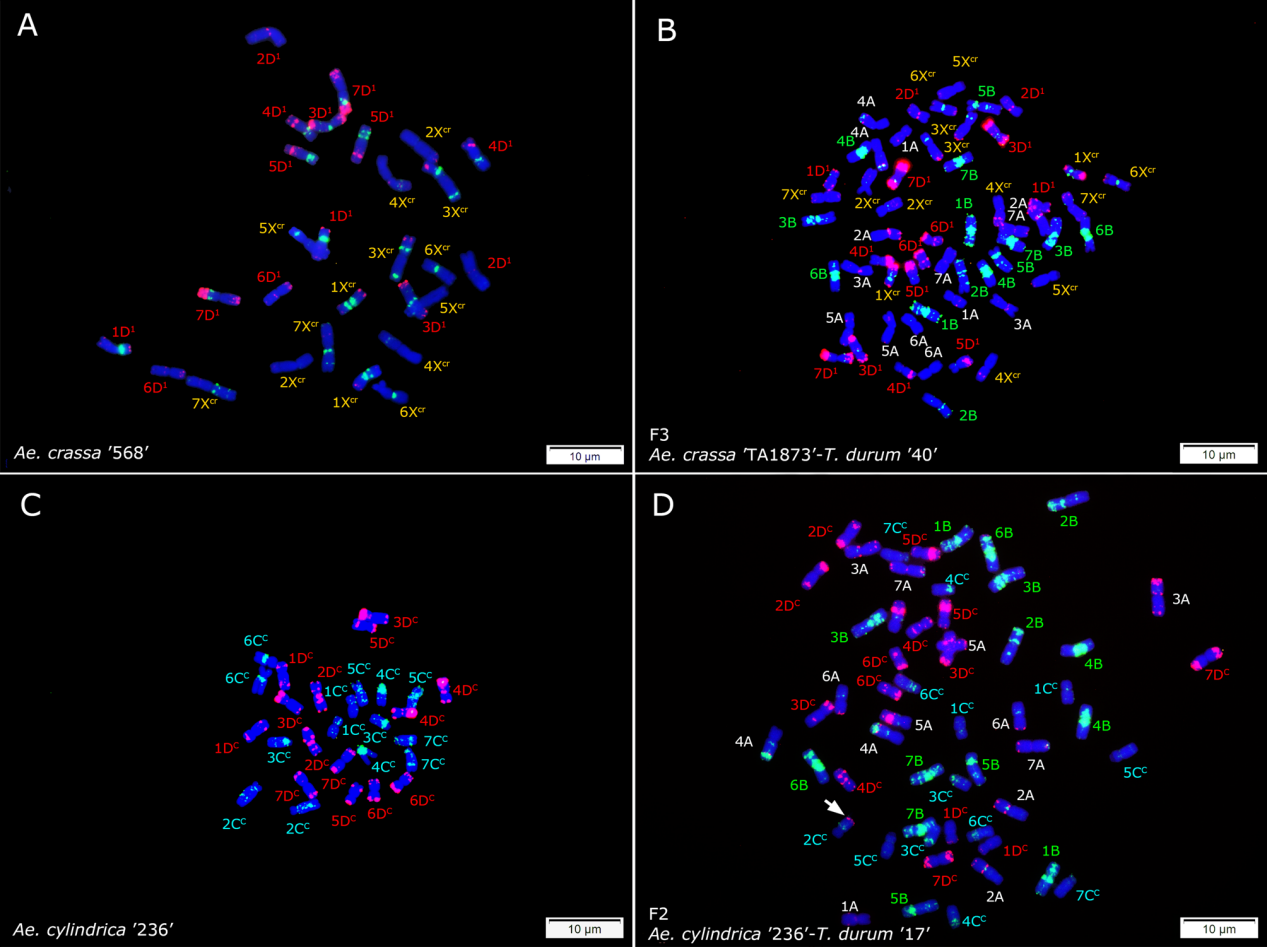
FISH signals from (GAA)_10_ (green) and p*Ta*535-1 (red) probes on mitotic metaphase chromosomes of *Ae. crassa* ‘AE 568’ with 2*n* = 4*x* = 28 chromosomes (**A**); an amphiploid from a cross between *Ae. crassa* ‘TA1873’-*T. durum* ‘40’ with 2*n* = 8*x* = 56 chromosomes (**B**); *Ae. cylindrica* with 2*n* = 4*x* = 28 chromosomes (**C**) and a metaphase cell of a monosomic amphiploid plant from a cross between *Ae. cylindrica* ‘236’-*T. durum* ‘17’ with 2*n* = 8*x* = 55 chromosomes where the 2C^c^ chromosome shows deletion/translocation (arrow) (**D**). Chromosomes were conunterstained with DAPI (blue).

FISH allowed to identify all the chromosomes in the *Triticum* and *Aegilops* parental species, hybrids and F_2_ lines, with reference to the chromosome length, arm ratio, and p*Ta*535-1 and GAA banding pattern parameters (Fig. 5, 6, 7). p*Ta*535-1 oligonucleotide probe mainly hybridized with the A- and D-genome chromosomes in combination with the GAA probe. C-banding and FISH using GAA-oligonucleotide probes on accession 49667 of *T. dicoccum* showed a general agreement in banding patterns (Fig. 5A, Supplementary Fig. S4). Although C-banding generally revealed more bands, banding patterns of both methods were generally similar, confirming that GAA microsatellite loci colocalize with C-bands in the genus *Triticum*.

Cytogenetic analysis also unexpectedly revealed that accession ‘AE 1211’ of *Ae. tauschii* was autotetraploid with 2*n* = 4*x* = 28 chromosomes (Fig. 5B); interestingly, this accession was also one of the most fertile parents in the cross with emmer wheat. Based on GAA and p*Ta*535-1 banding patterns, a balanced reciprocal translocation involving chromosomes 5A^t^ and 6G was identified in *T. timopheevii* accession ‘131212’ resulting in 5A^t^S.6GL and 6GS.5A^t^L translocated chromosomes (Fig. 5C). This translocation was also observed in an amphiploid produced from a cross between this line and *Ae. tauschii* ‘AE 1602’ (Fig. 5F). Some chromosomal rearrangements or translocations were identified that are induced by polyploidization in the evaluated synthetic amphiploids, including one small heterozygous deletion at the distal end of the 1BL chromosome arm in *T. durum* ‘78’ × *Ae. tauschii* ‘191’ line (Fig. 6D) and a single 2C^c^ chromosome showing deletion/translocation in an amphiploid genotype from a cross between *Ae. cylindrica* ‘236’-*T. durum* ‘17’ (Fig. 7D). This genotype was a monosome with 2*n* = 8*x* = 55 chromosomes. The monosomic status of this genotype was further confirmed by chromosome counting in nine different mitotic metaphase cells of this plant (Supplementary Fig. S7). We checked the chromosome number of two *Ae. ventricosa*-*T. turgidum* and four *Ae. crassa*-*T. turgidum* amphiploid seedlings. Six different mitotic metaphase cells from a single *Ae. ventricosa* ‘AE 1511’-*T. turgidum* ‘13’ amphiploid plant were checked and all showed 54 chromosomes (Supplementary Fig. S8), while all the checked cell of another single amphiploid seedling (i.e. *Ae. ventricosa* ‘AE 357’-*T. turgidum* ‘11’) showed 53 chromosomes (Supplementary Fig. S8). On the other hand, all the four *Ae. crassa*-*T. turgidum* amphiploids were complete disomic plants with 2*n* = 8*x* = 56 chromosomes (Supplementary Fig. S9), suggesting higher stability of these material compared to *Ae. cylindrica*-*T. durum* and *Ae. ventricosa*-*T. turgidum* amphiploids.

### Phenotypic diversity of the amphiploids

**Figure 8:**
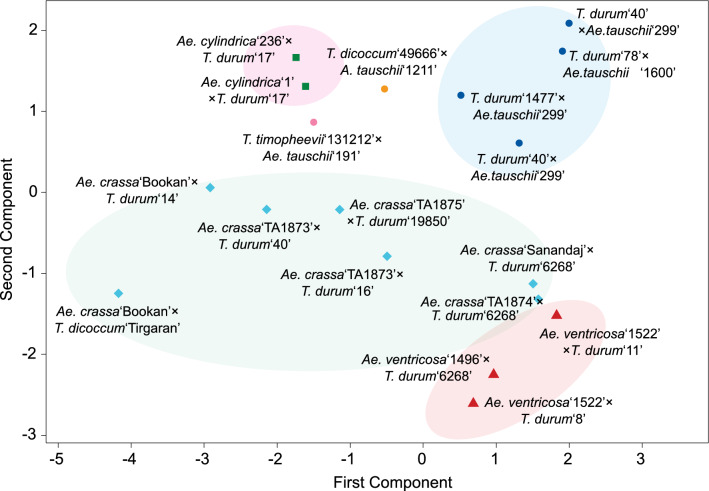
Principal component analysis (PCA) of the amphiploids from crossing between D-genome containing *Aegilops* species and *Triticum* (*T. durum*, *T. dicoccum* and *T. timopheevii*) genotypes based on the morphological traits of Table 1.

The produced amphiploids showed a high variation for most morphological traits measured (Table 1). We also observed a high level of diversity in spike morphology (Supplementary Fig. S5). Diversity was especially high for flag leaf width (5.6–19.6 mm), spikelets per spike (10.3–29.6; can also be seen in Fig. 2), spike length (11–22.4), seed Fe (19.2–56.1 µg/g DW) and Zn (14.2–60.2 µg/g DW) contents under no fertilizer conditions. Principal component analysis based on phenotypic traits grouped the amphiploids with the same genome composition together, although a higher variation was observed for the *Ae. crassa*-wheat amphiploids (Fig. 8). Amphiploids *Ae. cylindrica* ‘1’ × *T. durum* ‘17’, *Ae. ventricosa* ‘1522’ × *T. durum* ‘8’ and *Ae. crassa* ‘Sanandaj’ × *T. durum* ‘6268’ had high Fe and Zn contents in the seed.

**Table 1:** Morphological traits showing differences between some of the produced synthetic wheats and amphiploids.

Synthetic wheat or amphiploid	Tiller no.	Nodes No.	Pedancle length (cm)	Flagleaf width (mm)	Flagleaf length (cm)	Spiklete per spike	Spike length (cm)	Awn length (cm)	Plant height (cm)	Fe (µg/g DW)	Zn (µg/g DW)
**Genome: AABBDD**											
*T. durum* ‘40’ × *Ae. tauschii* ‘G 299-1’	18.6	2.0	44.5	12.2	34.1	20.6	13.3	2.1	90.4	26.5	16.0
*T. durum* ‘78’ × *Ae. tauschii* ‘AE 1600’	23.6	3.0	41.3	16.9	30.2	21.4	14.1	4.1	94.0	30.3	14.3
*T. durum* ‘40’ × *Ae. tauschii* ‘G 299-2’	33.6	3.0	43.8	18.9	27.2	20.4	14.6	5.0	96.0	30.3	17.1
*T. durum* ‘1477’ × *Ae. tauschii* ‘G 299’	38	3.0	33.0	17.0	28.5	20.0	12.0	6.5	84.0	38.7	30.1
**Genome: AAGGDD**											
*T. timopheevii* ‘131212’ × *Ae. tauschii* ‘AE 191’	44.4	2.0	35.8	12.9	15.5	17.3	14.6	6.1	60.8	37.9	38.6
**Genome: AABBDDDD**											
*T. durum* ‘49666’ × *Ae. tauschii* ‘AE 1211’	22.5	3.0	27.6	14.9	17.0	18.3	15.1	7.9	70.4	19.2	24.4
**Genome: D**^**1**^**D**^**1**^**X**^**cr**^**X**^**cr**^**AABB**											
*Ae. crassa* ‘Bookan × *T. durum* ‘14’	16.7	2.0	26.1	11.7	12.9	12.9	11.6	2.5	58.7	26.0	21.4
*Ae. crassa* ‘TA1873’ × *T. durum* ‘16’	8.8	2.0	28.6	22.4	26.2	15.5	15.6	3.7	64.2	31.7	20.9
*Ae. crassa* ‘Bookan’ × *T. dicoccum* ‘Tirgaran’	18.0	2.0	25.0	11.0	13.5	12.0	11.0	2.5	44.0	44.8	58.8
*Ae. crassa* ‘TA1874’ × *T. durum* ‘6268’	16.2	2.0	41.8	22.4	25.9	20.0	08.7	8.3	87.7	36.3	24.4
*Ae. crassa* ‘TA1875’ × *T. durum* ‘19850’	25.2	2.0	32.0	18.4	22.9	17.0	10.7	3.3	57.7	36.8	22.9
*Ae. crassa* ‘TA1873’ × *T. durum* ‘40’	16.7	2.0	31.1	12.7	13.4	13.1	12.1	3.6	69.9	33.0	25.0
*Ae. crassa* ‘Sanandaj’ × *T. durum* ‘6268’	19.7	2.0	46.2	17.7	24.3	19.0	17.5	9.5	93.5	56.1	39.6
**Genome: D**^**c**^**D**^**c**^**C**^**c**^**C**^**c**^**AABB**											
*Ae. cylindrica* ‘1’ × *T. durum* ‘17’	28.8	3.0	29.4	13.1	21.5	15.7	16.4	2.5	66.6	52.5	36.8
*Ae. cylindrica* ‘236’ × *T. durum* ‘17’	30.8	3.0	28.4	12.8	21.7	14.9	16.0	2.4	63.1	50.5	22.3
**Genome: D**^**v**^**D**^**v**^**N**^**v**^**N**^**v**^**AABB**											
*Ae. ventricosa* ‘AE 1522’ × *T. durum* ‘8’	16.4	2.0	37.0	19.0	34.4	17.0	13.2	12.0	77.6	44.9	60.2
*Ae. ventricosa* ‘AE 1496’ × *T. durum* ‘6268’	9.4	2.0	35.6	19.9	33.7	17.0	13.1	11.7	72.5	52.7	18.2
*Ae. ventricosa* ‘AE 1522’ × *T. durum* ‘11’	13.4	2.0	37.0	20.2	41.4	18.3	15.0	10.5	78.9	33.3	24.4

## Discussion

Global wheat production must substantially increase from its current level, to ensure food for a growing world population. This require continuous breeding activity and involvement of new and less exploited genetic resources. Here, in order to incorporate the genetic diversity of emmer wheat and exotic D genomes for the future wheat breeding, a collection of synthetic wheat lines and amphiploids were produced from crosses between tetraploid *Triticum* species and subspecies (AABB or AAGG genome) and the D genome containing species *Ae. tauschii* (diploids and one tetraploid with DD and DDDD genomes), *Ae. crassa* (D^1^D^1^X^cr^X^cr^ genome), *Ae. cylindrica* (D^c^D^c^C^c^C^c^ genome) and *Ae. ventricosa* (D^v^D^v^N^v^N^v^ genome) and amphiploids with AABBDD, AABBDDDD, AAGGDD, D^1^D^1^X^cr^X^cr^AABB, D^c^D^c^C^c^C^c^AABB and D^v^D^v^N^v^N^v^AABB genomes were generated. *Ae. crassa* has tetraploid and hexaploid cytotypes but tetraploid cytotypes were used in the present study (Fig. 7; Supplementary Fig. S9). We already have established FISH-based karyotypes of ‘Bookan’ and ‘Sanandaj’ accessions of this species^26^. Other D genome containing *Aegilops* species include *Ae. juvenalis* (Thell.) Eig (D^1^D^1^X^cr^X^cr^U^j^U^j^) and *Ae. vavilovii* (Zhuk.) Chennav. (D^1^D^1^X^cr^X^cr^S^v^S^v^) which were not used in the present study. However amphiploids from crossing durum wheat with these two species have been recently developed^34^. The produced amphiploids in the present study showed a high variation in morphological traits (Table 1). Based on phenotypic traits, amphiploids with the same genome composition grouped together in principal component analysis, although a higher variation was observed for the *Ae. crassa*-wheat amphiploids (Fig. 8).

We produced novel synthetic wheat lines using durum, *T. dicoccum* and *T. dicoccoides* wheat genotypes toward increasing the genetic diversity of all the bread wheat subgenomes. Highly variable emmer wheat and *Ae. tauschii* genotypes (as reflected by the spike morphologies in Fig. 2 and Supplementary file Figs. S1, S2) were used in the crosses. In total, 29 different synthetic hexaploid and octaploid F_2_ lines were recovered with AABBDD, AABBDDDD or AAGGDD genomes (Supplementary Spreadsheet S1). A high variation in the crossability rate among *Ae. tauschii* genotypes, with the highest interspecific crossability observed for G 299 and AE 1211 (the tetraploid accession) (Fig. 3). However, most F_1_ seeds recovered lacked an endosperm and required embryo rescue to germinate. In contrast, Ogbonnaya et al.^35^ found an *Ae. tauschii* genotype that produced endosperm-containing F_1_ seeds when crossed with wheat. In wheat × *Aegilops* crosses, wheat genotypes differ in their crossability with *Ae. tauschii*^36^. In fact, effect of major crossability genes in common wheat such as *Kr1* (5BL), *Kr2* (5AL), *Kr3* (5D) and *SKr* (5BS) is well known for the obtaining F_1_ hybrids^37,38^ and wheat genotypes with dominant alleles generally show less crossability. The presence of crossability genes in tetraploid forms of wheat has not been studied in detail. However, there are reports suggesting chromosomes 7A and 4B are involved, so the crossability in tetraploid and hexaploid wheat might be controlled by different genetic systems^39,40^.

Many synthetic hexaploids has been produced in CIMMYT, from crosses between durum wheat (*T. turgidum*) cultivars and *Ae. tauschii* accessions^23,41^. However, synthetic wheat production has mainly been confined to the crossing of durum wheat with *Ae. tauschii*. Although, emmer-based hexaploid lines has been developed directly from emmer wheat × hexaploid wheat crosses and backcrossing to hexaploid wheat^42,43^ and useful traits, such as protein content and test weight^42^ and water-use-efficiency of grain production^43^ was introduced from emmer to hexaploid wheat.

In addition to the synthetic wheat lines, 27 different octaploid F_2_ amphiploid lines were recovered from crosses between Tetraploid *Aegilops* species (i.e. *Ae. crassa*, *Ae. cylindrica* and *Ae. ventricosa*) as female parents and *T. turgidum* genotypes as male parents (Supplementary Spreadsheet S1). A high rate of endosperm containing plump seeds from tetraploid *Aegilops* species (♀) × tetraploid wheat (♂) crosses were produced in the present study (Fig. 3). The overall mean crossability in all the crosses between tetraploid *Aegilops* species (i.e. *Ae. crassa*, *Ae. cylindrica* and *Ae. ventricosa*) and *T. turgidum* was 0.51 which is apparently higher than the mean crossability recorded for *Triticum*-*Ae. tauschii* crosses (i.e. 0.062). Tetraploid and hexaploid *Aegilops* species generally show higher crossability than diploid *Aegilops* species when crossed with common or tetraploid wheat and generally tend to set endosperm more resulting in plump F_1_ seeds^44,45^. Spontaneous F_2_ seed production has been reported in *Ae. crassa* × *T. persicum* hybrids^21^. Delibes and Garcia-Olmedo ^46^ reported hybridization between *wheat* and *Ae. ventricosa*. Yuan, et al.^47^ reported the production of *Ae. cylindrica* × *T. aestivum* hybrids that showed 0 to less that 1% seed set in back-crosses. Occurrence of interspecific hybrids between *Ae. cylindrica* and *T. aestivum* has also been reported in the field^48^. Fakhri et al. also concluded that lack of F_2_ seed in reciprocal crosses between *T. aestivum* and *Ae. cylindrica* hybrids might be due to lack of meiotic restitution and low rate of viable gametes^19^. Xu and Dong^21^ also reported complete sterility of *Ae. cylindrica* × *T. persicum* hybrids. While it was very hard to cross tetraploid *Aegilops* species as the male parent to *T. turgidum* (Supplementary Fig. S6), the reverse cross direction was more successful in the present study.

We analyzed the rates of unreduced male gametes in 12 different hybrid plants and found a significant correlation between unreduced gamete and seed set. Hence, we believe that the F_2_ seeds are mainly the product of unreduced gametes rather than somatic chromosome doubling. While *Ae. cylindrica* has been reported to spontaneously cross with *T. aestivum* and frequently crossed with *T. aestivum* artificially, the resulting hybrid plants (2n = ABDD^c^C^c^) are sterile and do not produce viable gametes^19,49^. In our work, F_1_ plants from the crosses between *T. durum* (♀) and *Ae. cylindrica* (♂) were completely sterile and no F_2_ seeds were produced (Fig. 4G; Supplementary Fig. S6), while healthy F_1_ seeds were produced from reverse crosses. The germination rate of F_1_ seeds from *Ae. cylindrica* × *T. turgidum* was very low and only two different F_1_ plants (from two different cross types) reached to maturity and 6 F_2_ seeds were harvested (Supplementary Spreadsheet S1; Supplementary Fig. S5). These results confirm that crossability highly depends on parental species, their ploidy level and cross direction. Interestingly, we produced eight auto-allo-octaploid synthetic wheat line with AABBDDDD genome in F_2_ using a tetraploid *Ae. tauschii* as the male parent (Fig. 6C). But the stability of these line may be affected by their auto-allopolyploidy status during the next generations.

One small heterozygous deletion at the distal end of the 1BL chromosome arm in the F_2_ generation of *T. durum* ‘78’ × *Ae. tauschii* ‘AE 191’ line (Fig. 6D) and a monosomic amphiploid genotype from a cross between *Ae. cylindrica* ‘236’-*T. durum* ‘17’ (D^c^D^c^C^c^C^c^AABB genome) carrying a single rearranged 2C^c^ chromosome were identified (Fig. 7). No other chromosomal arrangement or translocation induced by polyploidization, was identified in the evaluated synthetic wheat lines implying their genome stability.

All the four evaluated *Ae. crassa*-*T. turgidum* amphiploids (D^1^D^1^X^cr^X^cr^AABB genome) showed a complete set of 2*n* = 8*x* = 56 chromosomes. Contrary to the *Ae. cylindrica*-*T. turgidum* F_2_ plants which set plump and shriveled seeds in less than 50% of the florets, *Ae. crassa*-*T. turgidum* amphiploids F_2_ plants were completely fertile and set plump healthy seeds. Naranjo and Benavente^50^ observed high levels of chiasmata in *Ae. crassa*-wheat hybrids with the *Ae. crassa* cytoplasm, suggesting that *Ae. crassa* cytoplasm induces homoeologous pairing. Such a cytoplasmic effect—if available in our *Ae. crassa*-*T. turgidum* amphiploids—may lead to chromosomal rearrangement in the next generation, however, no rearrangement was detected in an *Ae. crassa*-*T. turgidum* individual amphiploid analysed by FISH (Fig. 7B).

The two evaluated *Ae. ventricosa*-*T. turgidum* and one *Ae. cylindrica*-*T. turgidum* amphiploid individuals were aneuploids with 2*n* = 54 (Supplementary Fig. S8, 2*n* = 53 (Supplementary Fig. S8) and 2*n* = 55 (Supplementary Fig. S7) chromosomes. Such a chromosome elimination commonly results from production and union of partially unreduced gametes in F_1_ plants which is caused by meiotic irregularities such as uni- and multivalent formation and lagging chromosomes^44^. Chromosome loss may also happens in upcoming generations. The stability of amphiploids from the *Triticum* or *Aegilops* is also affected by parental species and genotype, the effect of specific genes and the rate of parental genome affinity^40,45,51–53^. However, chromosome elimination in offspring of high ploidy level amphiploids may lead to stabile partial amphiploids over subsequent generations^54^. More genomic analysis of the offspring is required to find out the genomic stability and transmission, because going through more rounds of meiosis would provide chance for possible genome rearrangements.

Crossing between the synthetic wheat and amphiploid lines produced in the present study with *T. aestivum* and repeated generations of self-pollination can generate bread wheat lines with recombined new subgenomes. In this way, new introgression lines with useful phenotypic traits can be recovered. Retention of individuals with only D chromosomes is also possible using marker assisted selection which results in new wheat lines with recombined new D subgenomes^18^.

## Conclusion

Here, a lot of crosses were made between tetraploid wheat (i.e. emmer wheat, *T. durum* and *T. timopheevii*) and the D-containing *Aegilops* species and successfully recovered various synthetic hexaploid and octaploid F_2_ lines with AABBDD, AABBDDDD, AAGGDD, D^1^D^1^X^cr^X^cr^AABB, D^c^D^c^C^c^C^c^AABB and D^v^D^v^N^v^N^v^AABB genomes via crossing, in vitro rescue of F_1_ embryos and spontaneous production of F_2_ seeds on the F_l_ plants. Diverse genotypes of emmer wheat and *Aegilops* species were used in the crosses and various forms of D subgenomes were brought together in the produced amphiploids. Contribution of D genome bearing *Aegilops* species and the less-investigated emmer wheat genotypes as parents in the crosses resulted in novel synthetic wheat and amphiploids which can further be used as bridges to expand the genetic variation of wheat beyond its current status via crossing and backcrossing.

## Supplementary information

Supplementary Figures.

Supplementary Information 1.

Supplementary Information 2.

## Data Availability

Seeds of the new synthetic wheat lines and amphiploids (indicated in the coloured cells of the last column in the Supplementary Spreadsheet S1) would be available for distribution via the Seeds and Plant Improvement Institute of Iran (SPII) after regeneration.
