# Supplementary material for: Production of synthetic wheat lines to exploit the genetic diversity of emmer wheat and D genome containing Aegilops species in wheat breeding
Source: Sci Rep. 2020 Nov 12;10:19698. doi: 10.1038/s41598-020-76475-7 (PMC7661528; doi:10.1038/s41598-020-76475-7)
Supplement: Supplementary file 1 — Supplementary Figures. [file 41598_2020_76475_MOESM1_ESM.docx]

Production of synthetic wheat lines to exploit the genetic diversity of emmer wheat and D genome containing *Aegilops* species in wheat breeding

Ghader Mirzaghaderi^1*^, Zinat Abdolmalaki^1^, Rahman Ebrahimzadegan^1^, Farshid Bahmani^1^, Fatemeh Orooji^1^, Mohammad Majdi^1^, Ali-Akbar Mozafari^2^

^*^ Corresponding author (Email: gh.mirzaghaderi@uok.ac.ir)

^1^ Department of Agronomy and Plant Breeding, Faculty of Agriculture, University of Kurdistan, P. O. Box 66177–15175, Sanandaj, Iran

^2^ Department of Horticultural Sciences, Faculty of Agriculture, University of Kurdistan, P. O. Box: 416, Sanandaj, Iran

**Supplementary file**

#### Correlations between morphological traits of emmer wheat genotypes. Range of each trait (min. and max.) based on mean data has been shown at the bottom of figure.

#### Clustered heat map of the standardized data from morphological traits showing differences between the emmer wheat genotypes which were used in the crosses with *Ae. tauschii.*


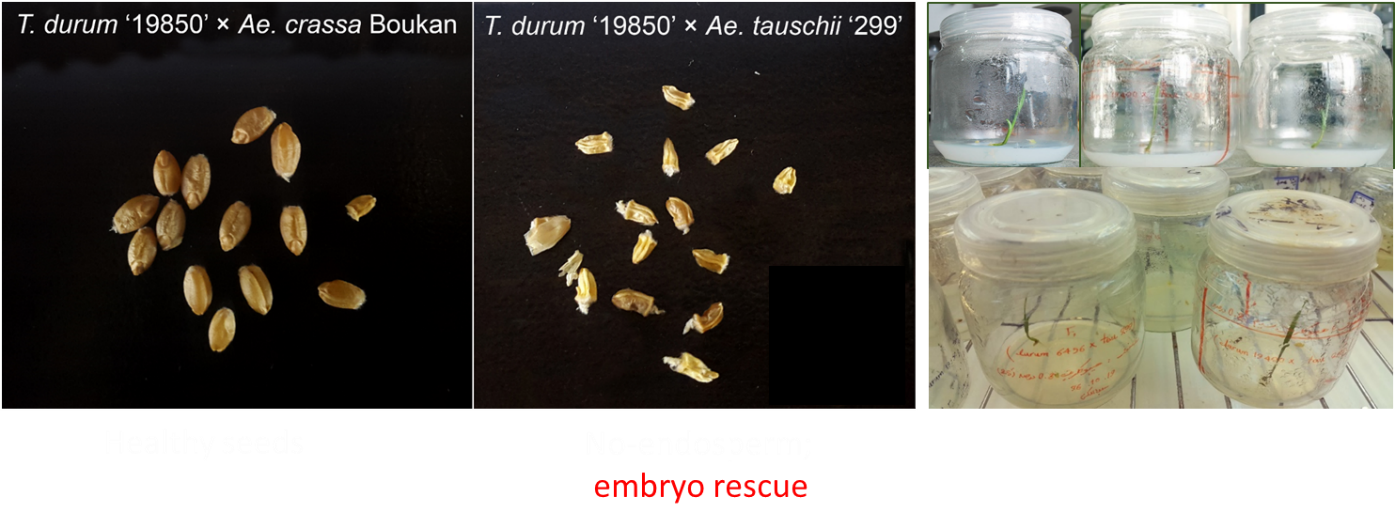


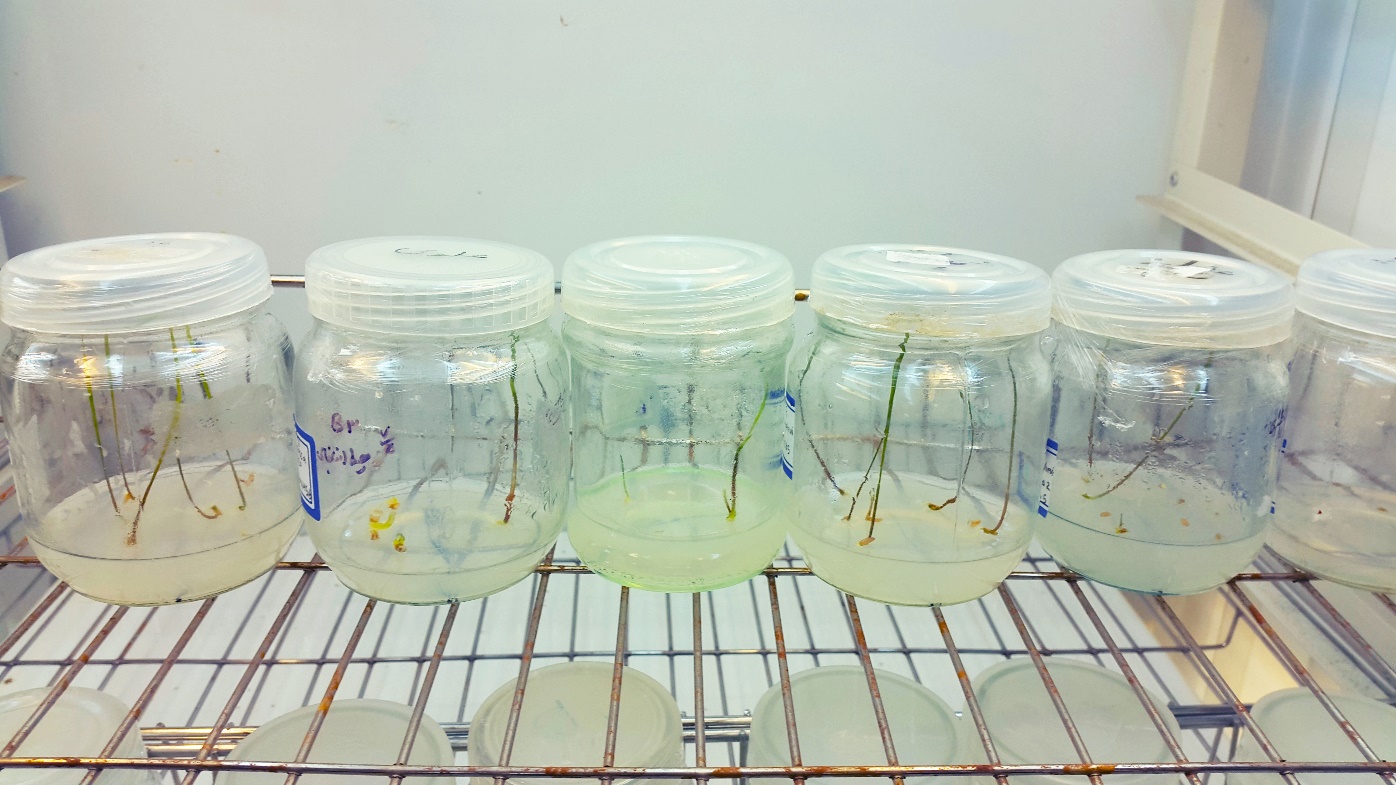


#### F_1_ seeds from crosses between tetraploid wheat and *Aegilops* species. Most of the F_1_ seeds from wheat and *Ae. tauschii* crosses lacked endosperm and required embryo rescue to germinate. On the other hand, most of the seeds from crosses between tetraploid *Aegilops* species (e.g. *Ae. crassa*) as female parents and wheat as male parents were plumped seeds that contained endosperm.


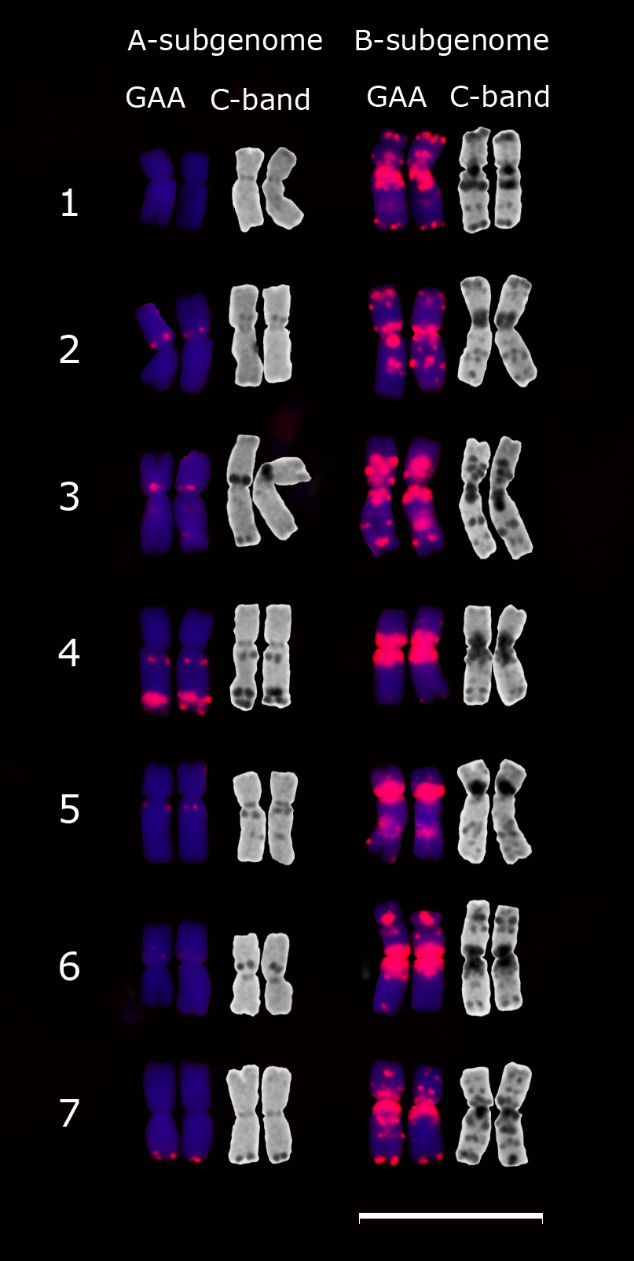


#### FISH using GAA-oligonucleotide probes and C-banding on accession 49667 of *T. dicoccum* showing a general agreement in banding patterns.


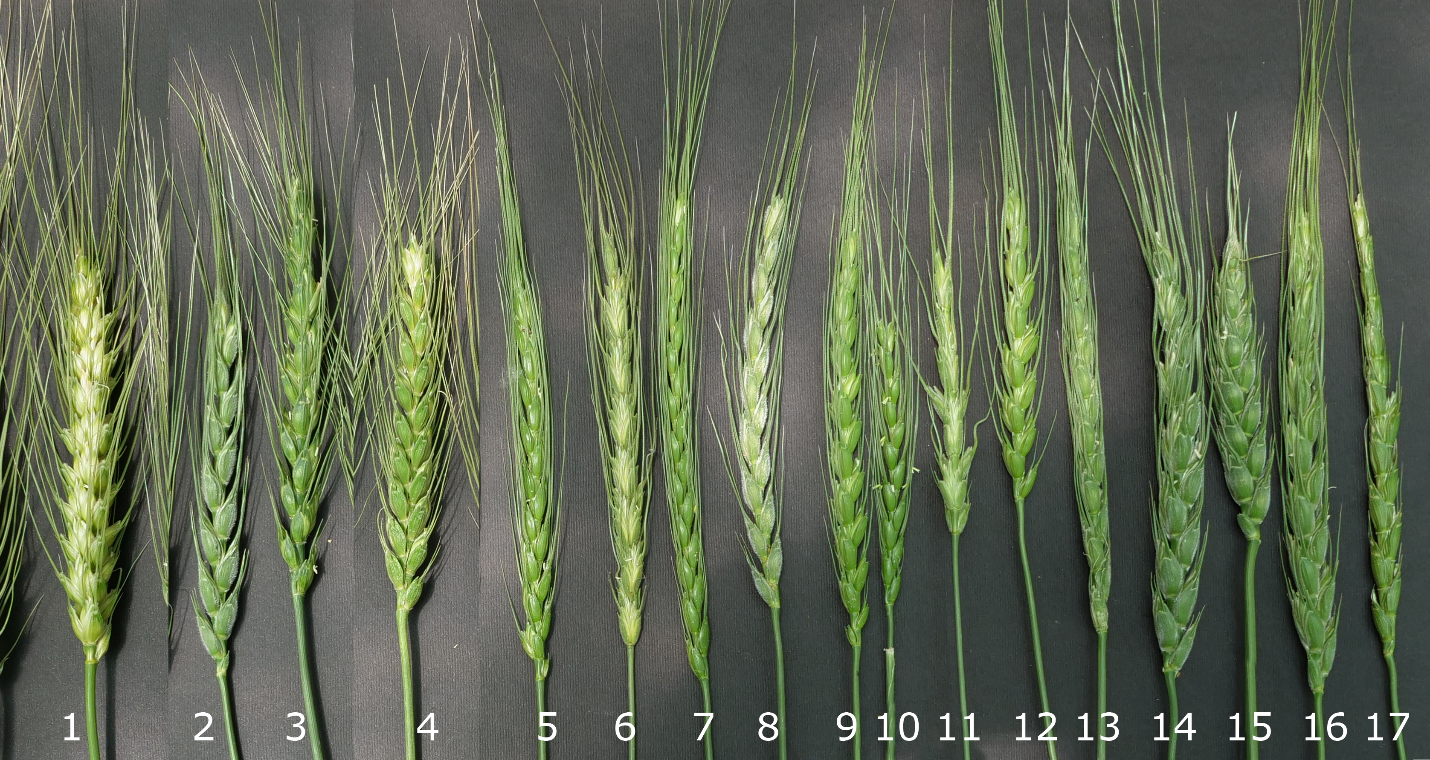


#### Spike morphology of a sample of amphiploids or BC_1_ lines from crosses between tetraploid wheat and *Aegilops* (*Ae. tauschii*, *Ae. crassa*, *Ae. ventricosa* and *Ae. cylindrica*) genotypes. **1**: F_2_ (*T. durum* ‘78’ × *Ae. tauschii* ‘600’); **2**: F_2_ (*Ae.* *crassa* ‘Bookan × *T. durum* ‘14’); **3**: F_2_ (*T. durum* ‘1477’ × *Ae. tauschii* ‘299’); **4**: F_2_ (*T. durum* ‘12595’× *Ae. tauschii* ‘13939’); **5**: F_1_ (*T. dicoccum* ‘IG-88753’ × *Ae. tauschii* ‘299’); **6**: F_1_ (*T. timopheevii* ‘131212’ × *Ae. tauschii* ‘1548’; **7**, **9**: F_1_ (*T. dicoccum* ‘IG-127691’ × *Ae. tauschii* ‘299’); **8**: F_1_ (*T. dicoccum* ‘49666’ × *Ae. tauschii* ‘1211’); **10**: F_1_ (*T. dicoccum* ‘Tirgaran’× *Ae. tauschii* ‘1211’); **11**: F_1_ (*T. dicoccum* ‘Bainjub’ × *Ae. tauschii* ‘1211’); **12**: F_1_ (*T. dicoccum* ‘Tirgaran’×At‘299’); **13**: F_1_ (*Ae.* *crassa* ‘Bookan’× *T. dicoccum* ‘Tirgaran’); **14**: F_2_ (*Ae.* *crassa* ‘Sanandaj’× *T. durum* ‘6268’); **15**: F_2_ (*Ae.* *crassa* ‘1873’× *T. durum* ‘40’); **16**: F_2_ (*Ae.* *crassa* ‘Bookan’× *T. durum* ‘6268’); **17**: F_2_ (*Ae. cylindrica* ‘l236’× *T. durum* ‘17’).


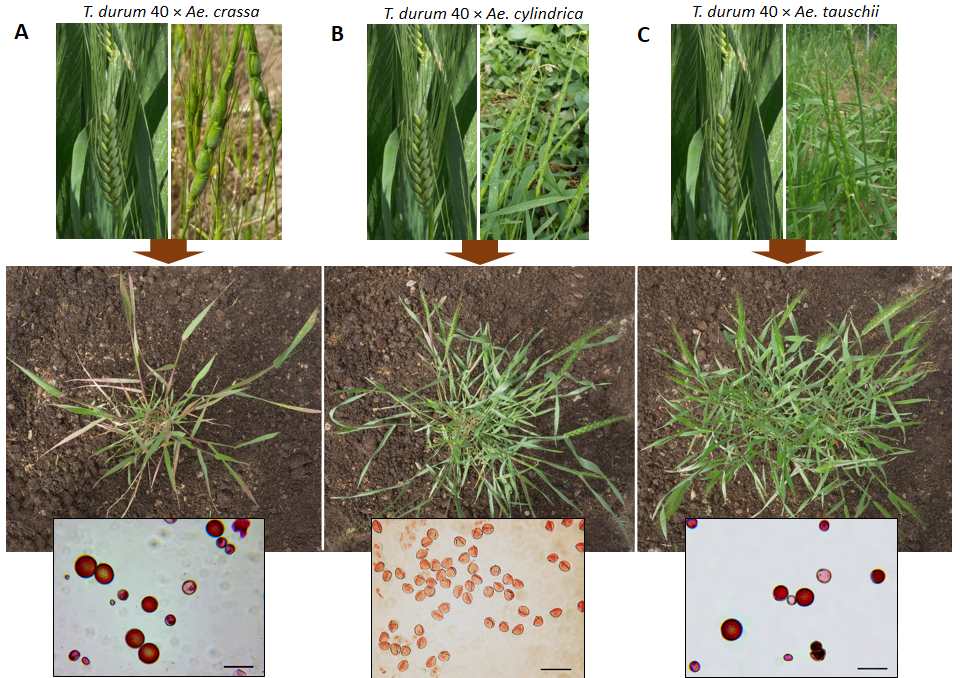


#### **A**) F_1_ ybrid plants from crosses between *T. durum* ‘40’ as female parent with *Ae. crassa* (**A**), *Ae. cylindrica* (**B**) and *Ae. tauschii* (**C**) as male parents. In cases A and B, a representative picture from its microspores shows both reduced (unviable) and unreduced (bigger and stainable) microspores. The hybrid plants from crosses between *T. durum* ‘40’ as female parent and *Ae. cylindrica* as male parent were sterile. The corresponding picture from their microspores indicates lack of viable pollen and compete sterility in this hybrid type.


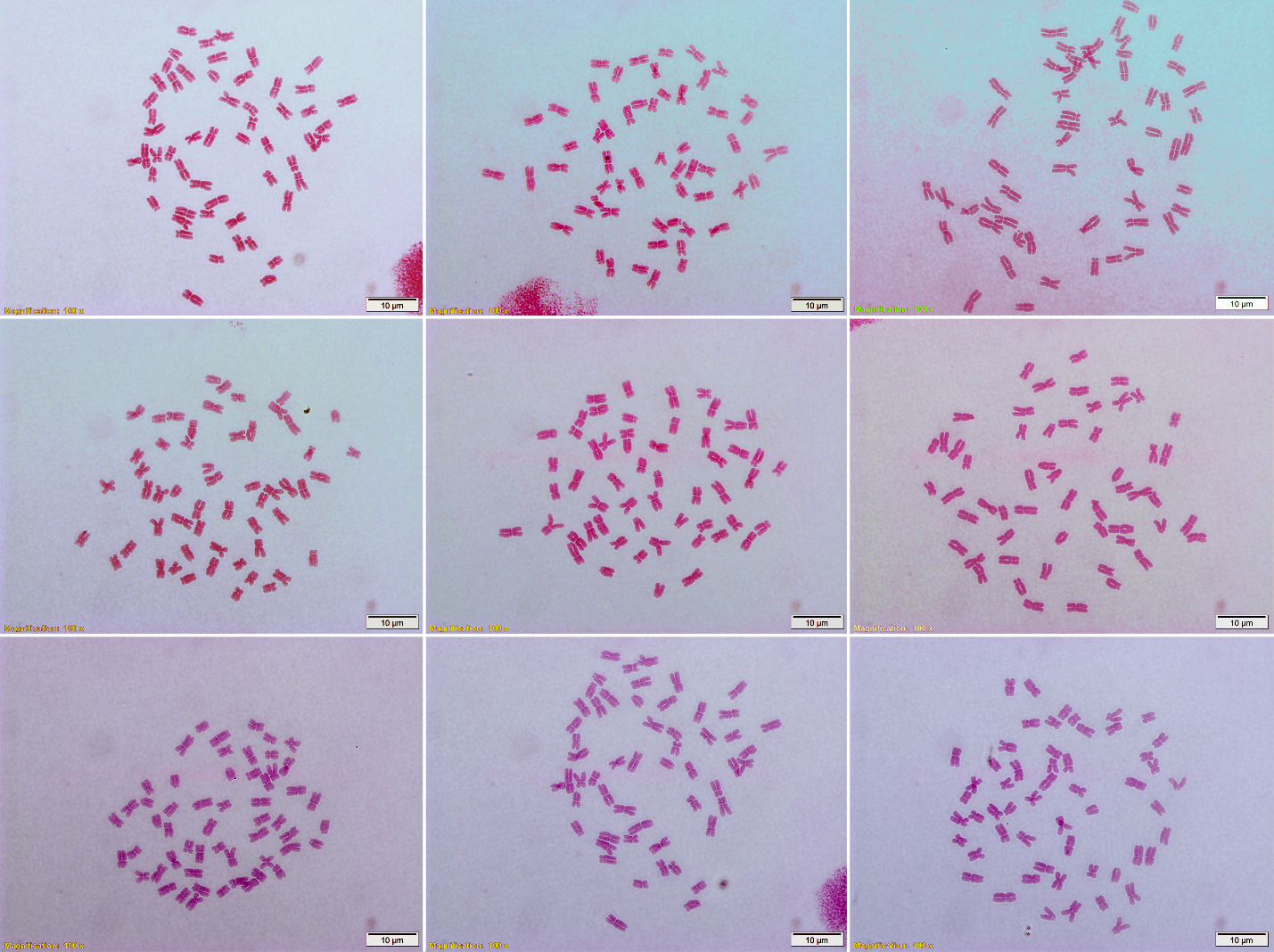


#### Acetocarmine stained mitotic metaphase chromosome spreads in nine different cells belonging to a single *Ae. cylindrica* ‘236’-*T. durum* ‘17’ amphiploid plant with D^c^D^c^C^c^C^c^AABB genome. All the spreads show 55 chromosomes indicating deletion of a single chromosome in this monosomic genotype.


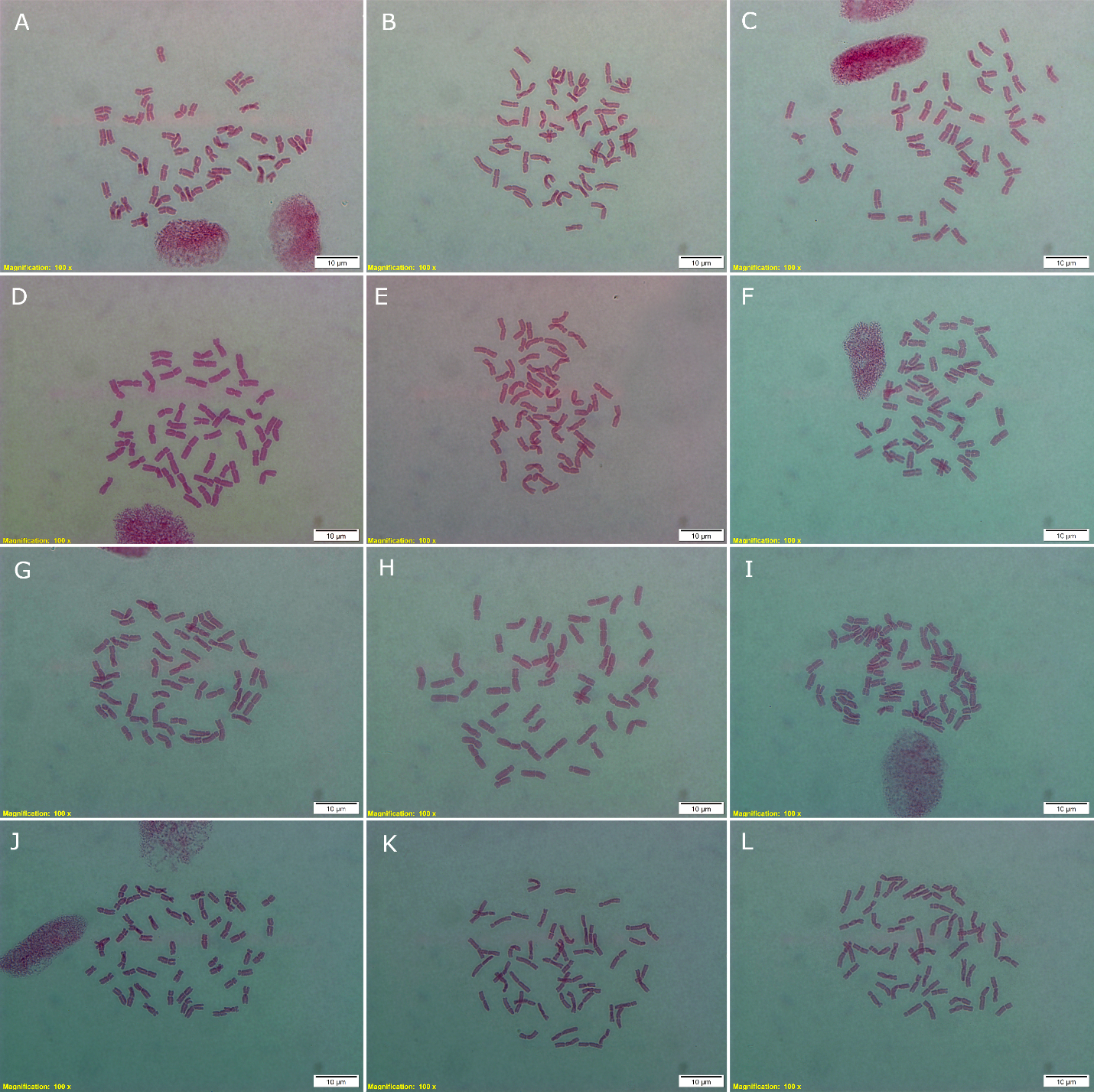


#### Acetocarmine stained mitotic metaphase chromosome spreads in *Ae. ventricosa* -*T. turgidum* amphiploids. A-F) Six different cells of a single *Ae. ventricosa* ‘1511’-*T. turgidum* ‘13’ amphiploid plant all showing 54 chromosomes. G-L) Six different cells of a single *Ae. ventricosa* ‘AE 357’-*T. turgidum* ‘11’ amphiploid plant all showing 53 chromosomes.


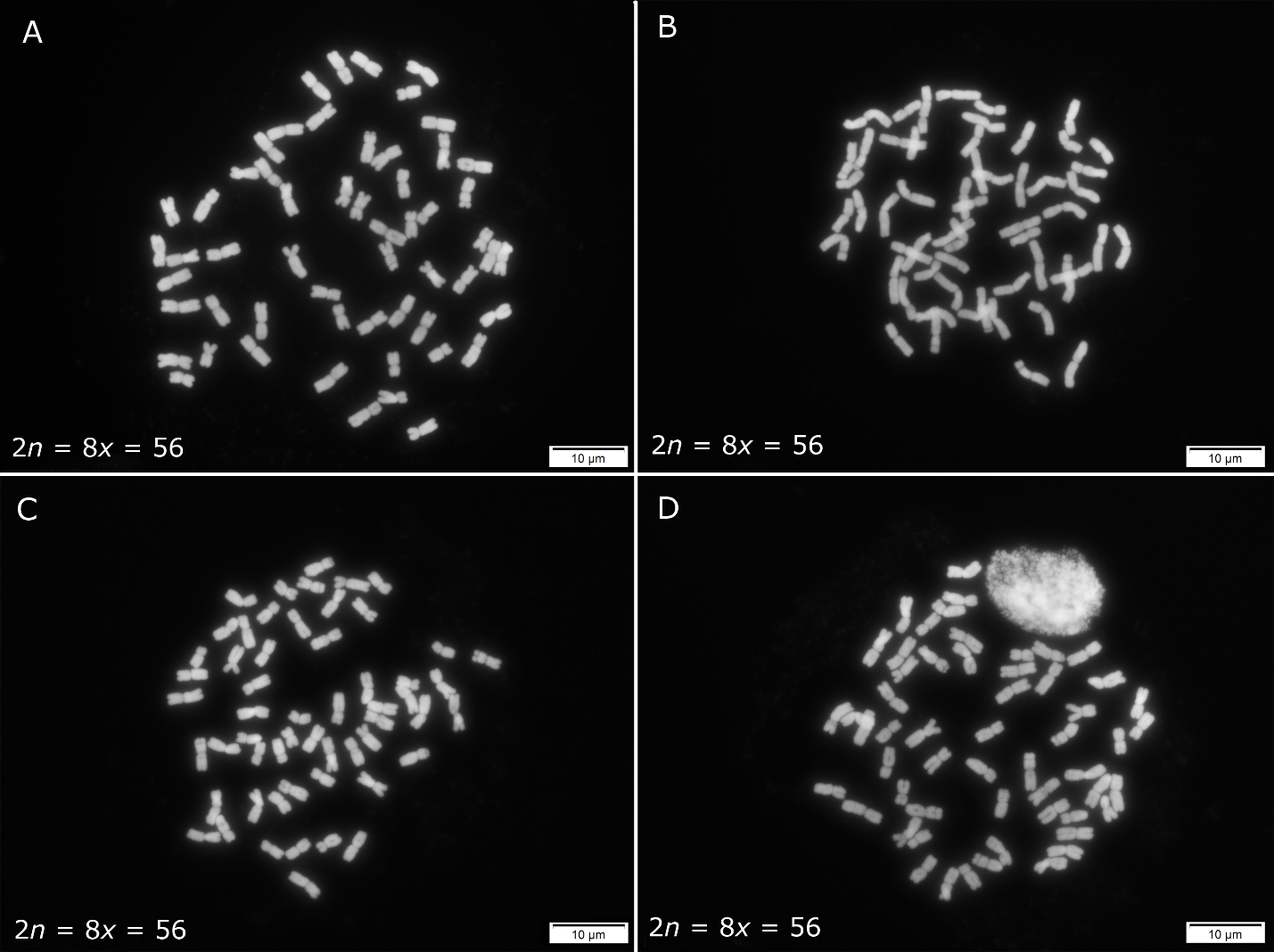


#### DAPI stained mitotic metaphase chromosome spreads of four different *Ae.* *crassa*- *T. durum* amphiploids show 2*n* = 8*x* = 56 chromosomes. **A**) *Ae.* *crassa* ‘Bookan × *T. durum* ‘14’, **B**) *Ae.* *crassa* ‘Sanandaj’× *T. durum* ‘6268’, **C**) *Ae.* *crassa* ‘TA1873’× *T. durum* ‘40’, **D**) *Ae.* *crassa* ‘TA1874’× *T. durum* ‘6268’.
